# The Hylemon-Björkhem pathway of bile acid 7-dehydroxylation: history, biochemistry, and microbiology

**DOI:** 10.1016/j.jlr.2023.100392

**Published:** 2023-05-19

**Authors:** Jason M. Ridlon, Steven L. Daniel, H. Rex Gaskins

**Affiliations:** 1 Department of Animal Sciences, University of Illinois Urbana-Champaign, Urbana, IL, USA; 2 Division of Nutritional Sciences, University of Illinois Urbana-Champaign, Urbana, IL, USA; 3 Carl R. Woese Institute for Genomic Biology, University of Illinois Urbana-Champaign, Urbana, IL, USA; 4 Cancer Center at Illinois, University of Illinois Urbana-Champaign, Urbana, IL, USA; 5 Center for Advanced Study, University of Illinois Urbana-Champaign, Urbana, IL, USA; 6 Department of Microbiology and Immunology, Virginia Commonwealth University School of Medicine, Richmond, VA, USA; 7 Department of Biological Sciences, Eastern Illinois University, Charleston, IL, USA; 8 Department of Biomedical and Translational Sciences, University of Illinois Urbana-Champaign, Urbana, IL, USA; 9 Department of Pathobiology, University of Illinois Urbana-Champaign, Urbana, IL, USA

**Keywords:** bile acids, intestinal lipid metabolism, gut microbiome, bile acid dehydroxylation, allo-bile acids, enterohepatic circulation

## Abstract

Bile acids are detergents derived from cholesterol that function to solubilize dietary lipids, remove cholesterol from the body, and act as nutrient signaling molecules in numerous tissues with functions in the liver and gut being the best understood. Studies in the early 20th century established the structures of bile acids, and by mid-century, the application of gnotobiology to bile acids allowed differentiation of host-derived “primary” bile acids from “secondary” bile acids generated by host-associated microbiota. In 1960, radiolabeling studies in rodent models led to determination of the stereochemistry of the bile acid 7-dehydration reaction. A two-step mechanism was proposed, which we have termed the Samuelsson-Bergström model, to explain the formation of deoxycholic acid. Subsequent studies with humans, rodents, and cell extracts of *Clostridium scindens* VPI 12708 led to the realization that bile acid 7-dehydroxylation is a result of a multi-step, bifurcating pathway that we have named the Hylemon-Björkhem pathway. Due to the importance of hydrophobic secondary bile acids and the increasing measurement of microbial *bai* genes encoding the enzymes that produce them in stool metagenome studies, it is important to understand their origin.

*The Ancients (amongst whom is Aristotle) thought it [bile]*

*to be a mere excrement, and to be of no other use than by*

*its acrimony to promote the excretion of the guts*.

-Thomas Gibson, The Anatomy of Humane Bodies Epitomized, (1682)

*Man's liver is a brownish blob*.

*That does a most prodigious job*.

*It manufactures gall, or bile*.

*And normally keeps some on file*.

*Stored neatly in a pear-shaped sac*.

*From there the liver's yields attack*.

*The food man eats, to change its state*.

*By methods man can't duplicate*,

*Or even halfway understand*.

*He ought to treat this outsize gland*,

*With due respect and loving care*.

*To keep it in top-notch repair*,

*Because to get along at all*.

*Man needs an awful lot of gall*.

-Irene Warsaw, JAMA, 1975, v. 231, p. 1260.

Despite many excellent recent reviews in the bile acid-microbiome field (1, 2, 3, 4, 5), so far, the historical developments leading up to our present mechanistic understanding of the microbial 7-dehydroxylation of bile acids have not been adequately covered (Fig. 1). Strikingly, derivatives of the hydrophobic secondary bile acid products of microbial 7-dehydroxylation, such as deoxycholic acid (DCA) and lithocholic acid (LCA), are, in general, preferred ligands for many host nuclear receptors (farnesoid X receptor (also known as *NR1H4*) (6, 7, 8), pregnane X receptor (or *NR1I2*) (9), vitamin D_3_ receptor (10), constitutive androstane receptor (or *NR1I3*) (11), retinoic acid receptor gamma T (12, 13), nuclear receptor 4A) (14) and G protein-coupled receptors (TGR5, (*GPBAR1*) (15, 16), muscarinic receptors (*CHRM2*, *CHRM3*) (17), sphingosine-1-phosphate receptor 2) (18) over the primary bile acids from which they derive. What has emerged in recent decades is that the ‘Western lifestyle’ of inactivity, diets low in fiber and high in processed carbohydrates and saturated fats, increases both the amount of bile entering the gastrointestinal (GI) tract and the hydrophobicity of the bile acid pool thereby affecting host metabolic function (3, 19) and increasing the risk of hepatobiliary and GI cancers in humans (2).

**Fig. 1:**
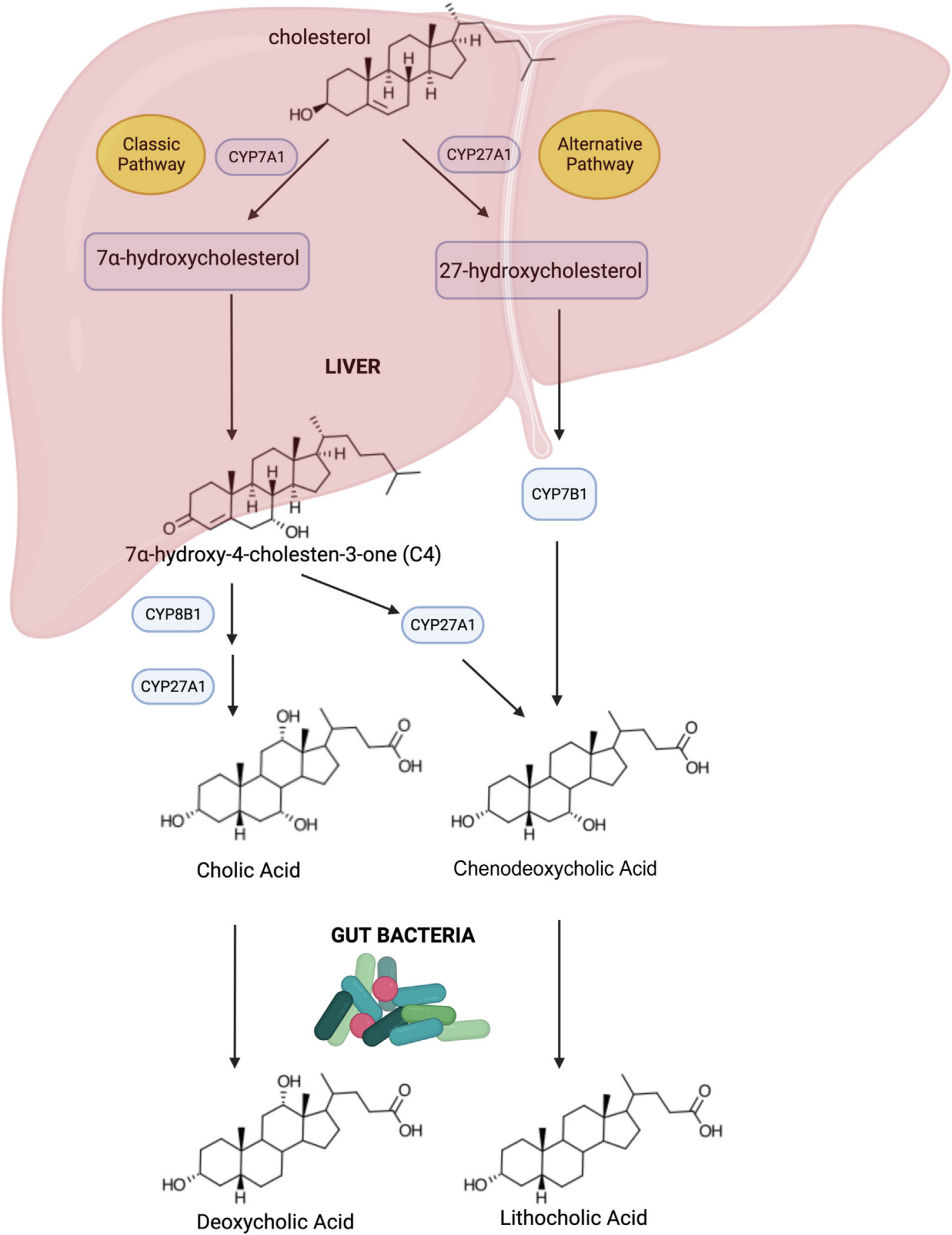
Bile salt biotransformations by intestinal bacteria. In the human liver, two primary bile acids, cholic acid (CA) and chenodeoxycholic acid (CDCA), are synthesized from cholesterol via the classic (neutral) pathway in hepatocytes. CDCA is also synthesized to a lesser degree by the alternative pathway. Bile acids are conjugated to taurine or glycine (not shown) in hepatocytes before secretion into the gallbladder. The intermediate, 7⍺-hydroxy-4-cholesten-3-one, is an important serum marker for BA synthesis, and it will later become apparent that its metabolism by intestinal bacteria provided important clues for the analogous 7⍺-dehydroxylation of CA and CDCA to deoxycholic acid (DCA) and lithocholic acid (LCA), respectively.

Thus, due to the importance of hydrophobic secondary bile acids and the increasing measurement of microbial genes encoding the enzymes that produce them (termed “bile acid inducible” or “*bai*” genes) in stool metagenome studies, it is important to understand how they came to be discovered and characterized. Here, we critically revisit this history and attempt to objectively identify the key researchers involved, the important observations made and to define the models of bile acid 7-dehydroxylation proposed during the 20th century, and how our knowledge has expanded into the 21st century. As a disclaimer and as Goethe warned, "Writing history is always a questionable thing. Despite best intentions one runs the risk of being unbalanced; indeed, whoever undertakes such a venture declares in advance that (s)he will cast some in the light and some in the shadow.” So, with that said, we apologize for any omissions of scientists and discoveries that we have made in this brief review of the history, biochemistry, and microbiology of bile acid 7-dehydroxylation.

## A brief history of bile acids

Notions of the biliary cycle, inspired by the feedback mechanism of blood circulation introduced by William Harvey, date back to the 17th century (20, 21). The introductory Gibson quote encapsulates the ancient view of bile prior to the paradigm shift of cycles and feedback that supplanted earlier notions of “flux and reflux” that date back to Aristotle and the idea of bile as a mere propellant of stool through the GI tract. Yet Western thinkers were starting to become skeptical of the wisdom of prior centuries. Indeed, Chinese materia medica was utilizing animal biles as early as the Zhou dynasty (c. 1046-256 BCE) (22). Johann Baptista van Helmont proclaimed bile as *viscus nobile et vitale* (23). It was Mauritius van Reverhorst who applied Harvey’s reasoning that only recirculation explains the large volume of daily blood flow to the biliary system in his description of a *motus bilis circularis*, an enterohepatic circulation of bile. In further departure from the Aristotelian influence, Reverhorst experimentally confirmed his hypothesis in 1691 by cannulating the bile duct of a dog and measuring with respect to time (a recent Galilean invention), the volume of collected bile in a living animal (24). From this experiment, it was clear that the amount of bile entering the small bowel far exceeded the amount excreted in feces. Reverhorst reasoned there were two fractions, an “earthy” sediment found in the feces and a “volatile” fraction that passes through “pores” in the intestine that enters the circulatory system and returns to the liver. We now identify these “pores” as high affinity transmembrane transport proteins expressed in the apical and basolateral surfaces of ileocytes (25).

The early attempts at chemical analysis of bile read more like alchemy (to us today) but, by contrast, were ultimately successful. Treatment of bile with lead acetate resulted in separation of two substances designated “bile resin” and “picromel” (a colorless, bittersweet fluid obtained from the lead acetate filtrate) or as Berzelius termed them “choleic acid” and “bilin” (26). It was Demarcay, an associate of Justus von Liebig, who first coined the term “bile acid” to denote the nitrogen-free acidic fraction of bile obtained by alkali treatment (27). Strecker identified two acidic components in ox bile, one containing nitrogen (glycocholic acid) and the other with nitrogen and sulfur (TCA) and deduced the correct chemical formula C_24_H_40_O_5_ for cholic acid (CA) (28). Subsequently, Mylius performed elemental analysis on another bile acid isolated from ox bile and termed it “DCA” due to it being one oxygen less than CA (29). LCA was later purified from gallbladder stones (30) and chenodeoxycholic acid (CDCA, Greek χήν (chen), "goose") from goose bile roughly a decade later (31). A wealth of information on the early chemistry of bile acids is reviewed in detail elsewhere (26, 32, 33, 34, 35).

Adolf Windaus and Heinrich Wieland were awarded the 1927 and 1928 Nobel Prize in Chemistry, respectively, for their laborious work on probable (although ultimately incorrect) structures shared by steroids, including cholesterol and bile acids (32). During the 1930s, the X-ray crystallographic work by Desmond Bernal explored the correct dimensions for ergosterol, cholesterol, and vitamin D (36). Bernal determined that sterols form double layers, with long, thin molecular dimensions more in line with elongated long-chain alcohols. This was inconsistent with the Wieland-Windaus formula that implied a compact molecular structure in which the ABC rings meeting at C9 (32). Subsequent experimental studies arrived at the now accepted cyclopentanoperhydrophenanthrene sterol nucleus with the five carbon side chain of bile acids that was subsequently confirmed by total chemical synthesis (21).

Collectively, this work fundamentally revolutionized our understanding of the constituents of one important fraction of bile and the structural relation of bile acids to cholesterol. What was yet unclear is the origin of each bile acid, their building blocks, and how their structures change during transit through the mammalian GI tract, a habitat densely populated by microbes.

## The establishment of “secondary” bile acids

**Fig. 2:**
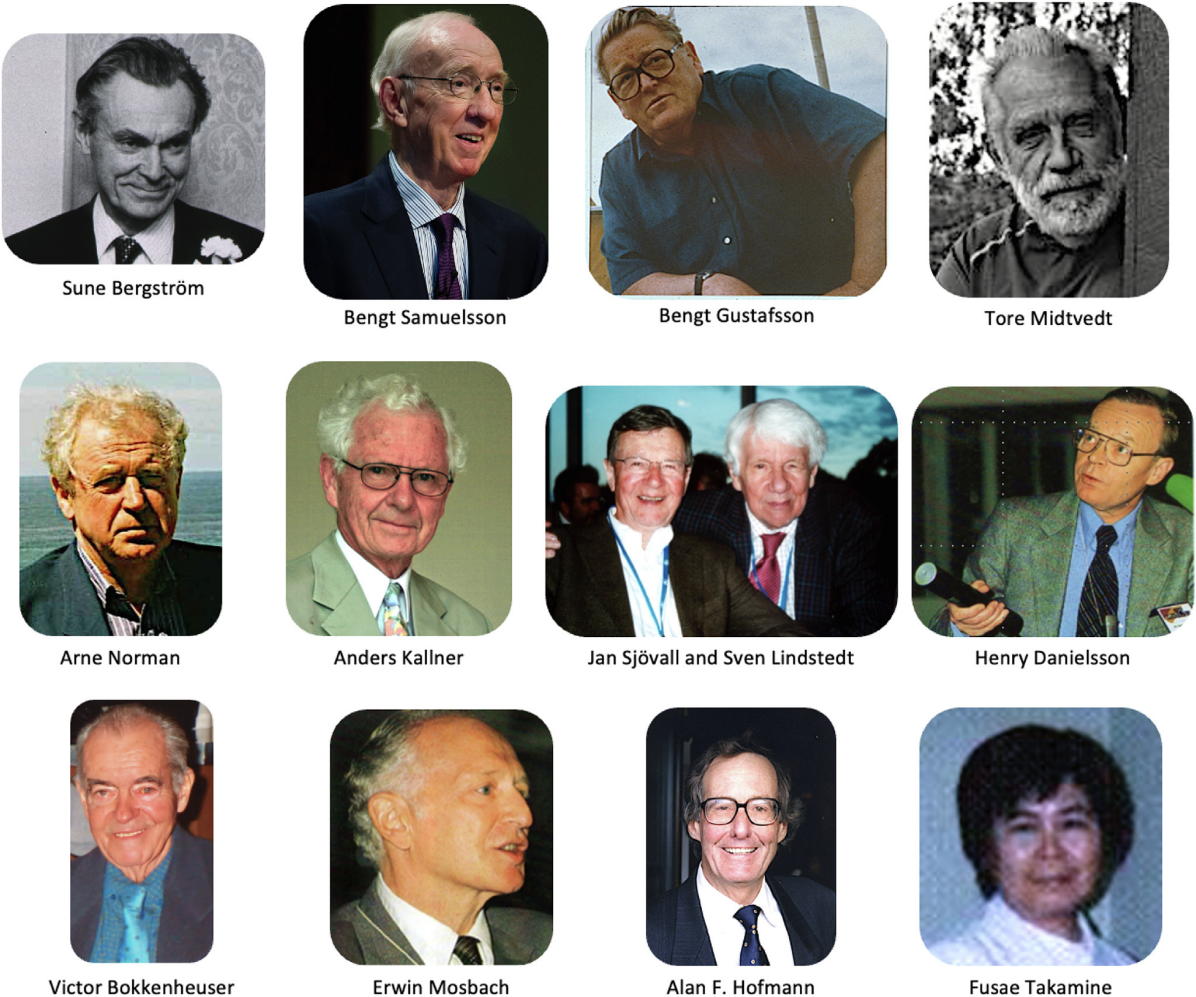
A selection of prominent researchers in the field of bile acid biochemistry and microbiology. (See text for descriptions and acknowledgments for photo permissions).

Starting in the 1930s, evidence was accumulating that bile acids were metabolized by intestinal bacteria. While proposed as early as 1896, it was Frankel in 1936 who first reported the in vitro hydrolysis of conjugated bile acids by animal and human intestinal content (37). Work by Schmidt and colleagues at Christ Hospital in Cincinnati with guinea pigs led to the identification of CA metabolites in the cecum, in cecal suspensions, and by a guinea pig cecal isolate of *Alcaligenes faecalis* which converted CA to tri-keto CA under aerobic conditions (38). During the 1950s and 1960s, Sune Bergstrӧm, Bengt Samuelsson, Jan Sjövall, Arne Norman, Sven Lindstedt, Anders Kallner, Henry Danielsson, Bengt Gustafsson, and Tore Midtvedt (Fig. 2), together with a team of talented coworkers, first at the University of Lund and later at the Karolinska Institutet in Sweden, would go on to make fundamental contributions to bile acid chemistry and biology (21, 39). Key to these discoveries was the nascent development of radiolabeling biochemicals, chromatographic methods to separate them, automatic liquid scintillation counters to quantify them, anaerobic microbial culturing to obtain pure cultures of bacteria responsible for metabolizing them, and gnotobiology to determine causation between microbe and bile acid metabolite (21, 39, 40).

Cholesterol was first labeled with deuterium in 1943 by the Nobel laureate, Konrad Bloch, who made a direct metabolic relationship between cholesterol and bile acid formation in an animal model (41). Soon after, [4-^14^C] and [26-^14^C]cholesterol began to be synthesized, allowing fundamental studies on the major pathways of cholesterol degradation in the liver of several mammalian species, including humans (39). In 1953, Bergstrӧm and Norman administered [4-^14^C]cholesterol to rats, observing formation of [4-^14^C]TCA in bile and multiple unconjugated derivatives of CA in the feces (42). That same year, Bergstrӧm and colleagues then developed a C-14 method to directly label bile acids at the C24 (carboxyl) position (43). In 1955, Lindstedt and Norman injected rats intraperitoneally with [24-^14^C]CA and [24-^14^C]CDCA and observed a complex pattern of bile acid metabolites, identical to those observed following administration of [4-^14^C]cholesterol (42, 44). Norman (1955) administered [24-^14^C]CA to conventional rats and rats treated with antibiotics and concluded that the [24-^14^C]CA was converted to [24-^14^C]TCA in the liver and deconjugated by bacteria in the intestine (45, 46).

In 1958, Norman and Sjövall administered [24-^14^C]CA and [24-^14^C]7-keto-DCA into rats and observed that bile acid hydrolysis occurs in the cecum, along with DCA formation (47). It was not clear whether 7-keto-DCA is an intermediate in the formation of DCA, or in equilibrium with CA, but this work established that both 7-keto-DCA and DCA are microbial products of CA. The results of similar experiments demonstrated that LCA is a microbial product of the 7-dehydroxylation of CDCA (48). In 1960, Norman demonstrated the conversion of CA to DCA after anaerobic culturing of mixed rat cecal microbiota (49). Two years later, Norman and Shorb reported the conversion of [24-^14^C]CA and [24-^14^C]CDCA by anaerobic suspensions of human stool bacteria (50). They suggested that the insolubility of LCA in aqueous culture may explain the relatively low levels of LCA observed in the bile relative to DCA, which must be absorbed into portal circulation more efficiently. Based on these collective observations, Bergström, Danielsson, and Samuelsson would suggest the use of the terms “primary” bile acid (principally C7-hydroxylated) as host derived and “secondary” bile acid as microbial conversion products (originally narrowly implied as C7-dehydroxylated) (39).

Another important technology was being developed in parallel to determine the function of host-associated microbes. Louis Pasteur believed commensals were absolutely required for multicellular life, while Marceli Nencki and Élie Mechnikov argued that vigor could be enhanced and life prolonged without contaminating and toxin-producing microorganisms (51, 52, 53). Over a ten-year period, starting in 1885, germ-free (GF) guinea pigs were delivered and maintained on sterile milk, settling the question that life was possible without commensal microbes (54, 55, 56).

It was only natural for additional questions to be raised, spurring improvements in technology to determine the role of host-associated microbiota in animal physiology and metabolism. A stainless steel, autoclavable gnotobiotic isolator for the rearing of GF rats was reported in 1946 by Bengt Gustafsson in Sweden (57) and the same year by Reyniers and coworkers in the USA (58). In 1957, Gustafsson, Bergström, Lindstedt, and Norman showed that [24-^14^C]CA administration to GF rats resulted in fecal excretion of [24-^14^C]TCA, although monocolonization with a strain of *Clostridium perfringens* resulted in deconjugation and accumulation of [24-^14^C]CA in feces. The bile acid pool size in GF rats was notably larger than that in conventional rats, despite lower daily fecal excretion (59), an observation only recently explained through the antagonistic effects of tauro-β-muricholic acid on farnesoid X receptor signaling (60).

The work of Gustafsson, Midtvedt, and Norman in the 1960s comparing bile acids in intestinal content between conventional and GF rats conclusively showed that microbes biotransform bile acids in vivo (61, 62). Monocolonization of GF rats with *Escherichia coli* resulted in the appearance of 7-oxoDCA, indicating the presence of bile acid 7ɑ-hydroxysteroid dehydrogenase (7ɑ-HSDH) activity (63); the gene encoding bile acid 7ɑ-HSDH and the structure was identified many decades later (64, 65). The strictly anaerobic bile acid 7ɑ-dehydroxylating bacteria had evaded isolation up to 1960 when Samuelsson and Bergström turned to clever radiolabeling strategies that led to a proposed mechanism by which the C7 hydroxyl group is removed by gut microbiota.

## The Samuelsson-Bergström model of bile acid 7ɑ-dehydroxylation

**Fig. 3:**
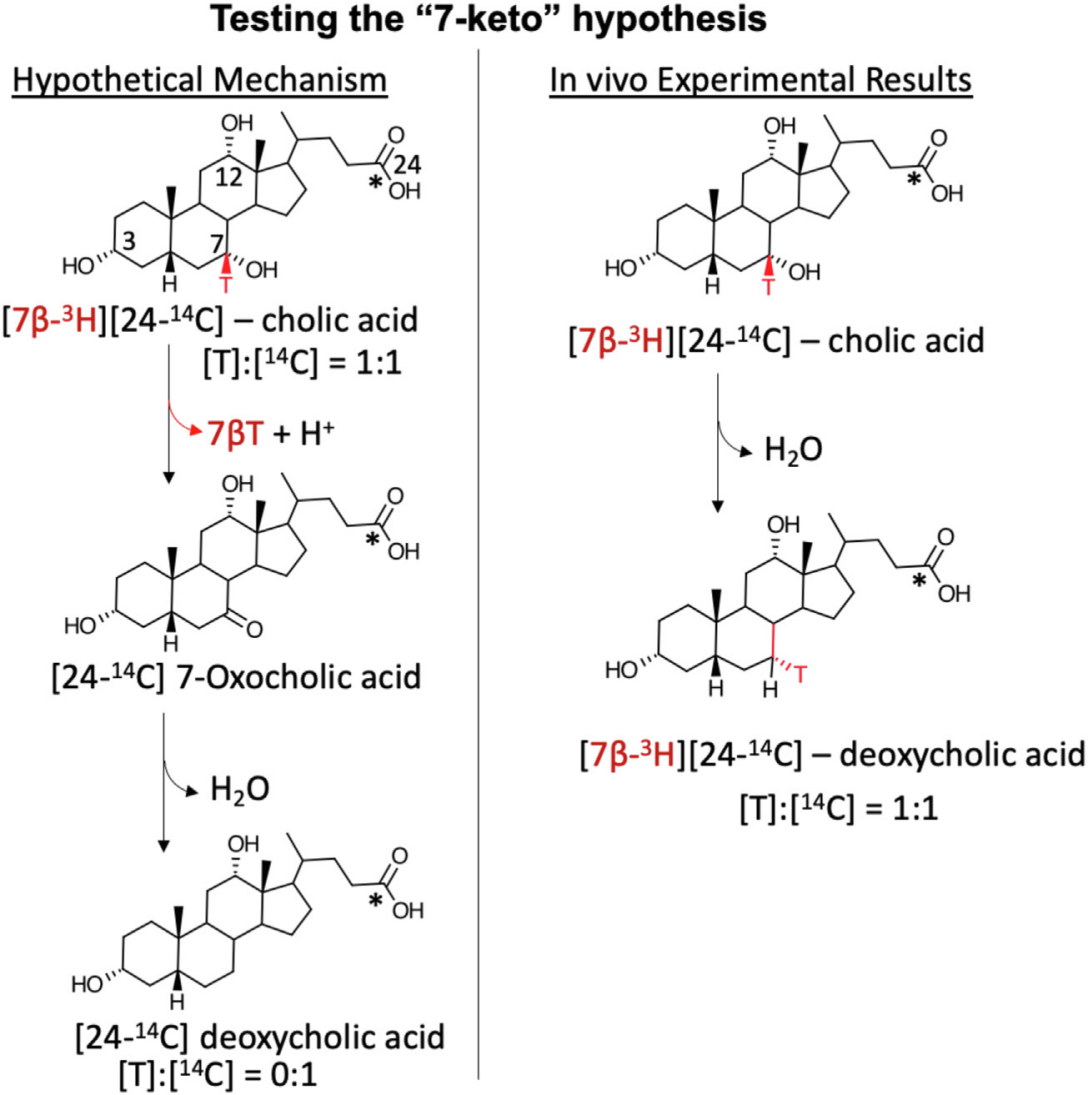
Testing the “7-keto-intermediate” hypothesis. Early experiments in rodent models observed the conversion of radiolabeled CA to both 7-oxoDCA and DCA. It was hypothesized that 7-oxoDCA may serve as an intermediate between CA and DCA. To test this hypothesis, ^3^H was added to [24-^14^C]CA at the C7 position. If 7-oxoDCA was an intermediate, it was expected that after in vivo metabolism of [7β-^3^H][24-^14^C]CA ([^3^H]:[^14^C] ratio = 1:1), fecal extraction of the end products should yield a [^3^H]:[^14^C] ratio of 0:1, indicating a loss of the [7β-^3^H]. However, the experimental results yielded DCA with a [^3^H]:[^14^C] ratio of 1:1, indicating that 7-oxoDCA is *not* an intermediate in the bile acid 7⍺-dehydroxylation pathway. DCA, deoxycholic acid.

Early work had established that 7-oxoDCA is formed from CA in the intestine of rats (47) and rabbits (66). Administration of either [24-^14^C]CA or [24-^14^C]7-oxoDCA to rats resulted in the formation of [24-^14^C]DCA. Two possibilities were proposed, the first is that bile acid 7ɑ-dehydroxylation may proceed through a 7-keto-DCA intermediate. Alternatively, 7-keto-DCA was shown to be converted to CA by bacterial enzymes which would then be 7ɑ-dehydroxylated to DCA (66, 67). Formation of a 7-oxoDCA intermediate through dehydrogenation proceeds via removal of the 7ɑ-hydroxy proton and 7β-hydrogen. The “7-keto-intermediate” hypothesis was thus tested by observing the fate of the tritium label during metabolism of [24-^14^C][7β-^3^H]CA in vivo (Fig. 3). The “7-keto-intermediate” hypothesis was disproved after it was observed in experiments that no change occurred in the ratio of [^3^H]:[^14^C] in DCA (as [24-^14^C][7ɑ-^3^H]DCA) recovered from intestinal contents when compared to the ratio of [^3^H]:[^14^C] recovered in CA (as [24-^14^C][7β-^3^H]CA), indicating the retention of the tritium at C7 in the formation of DCA (66).

**Fig. 4:**
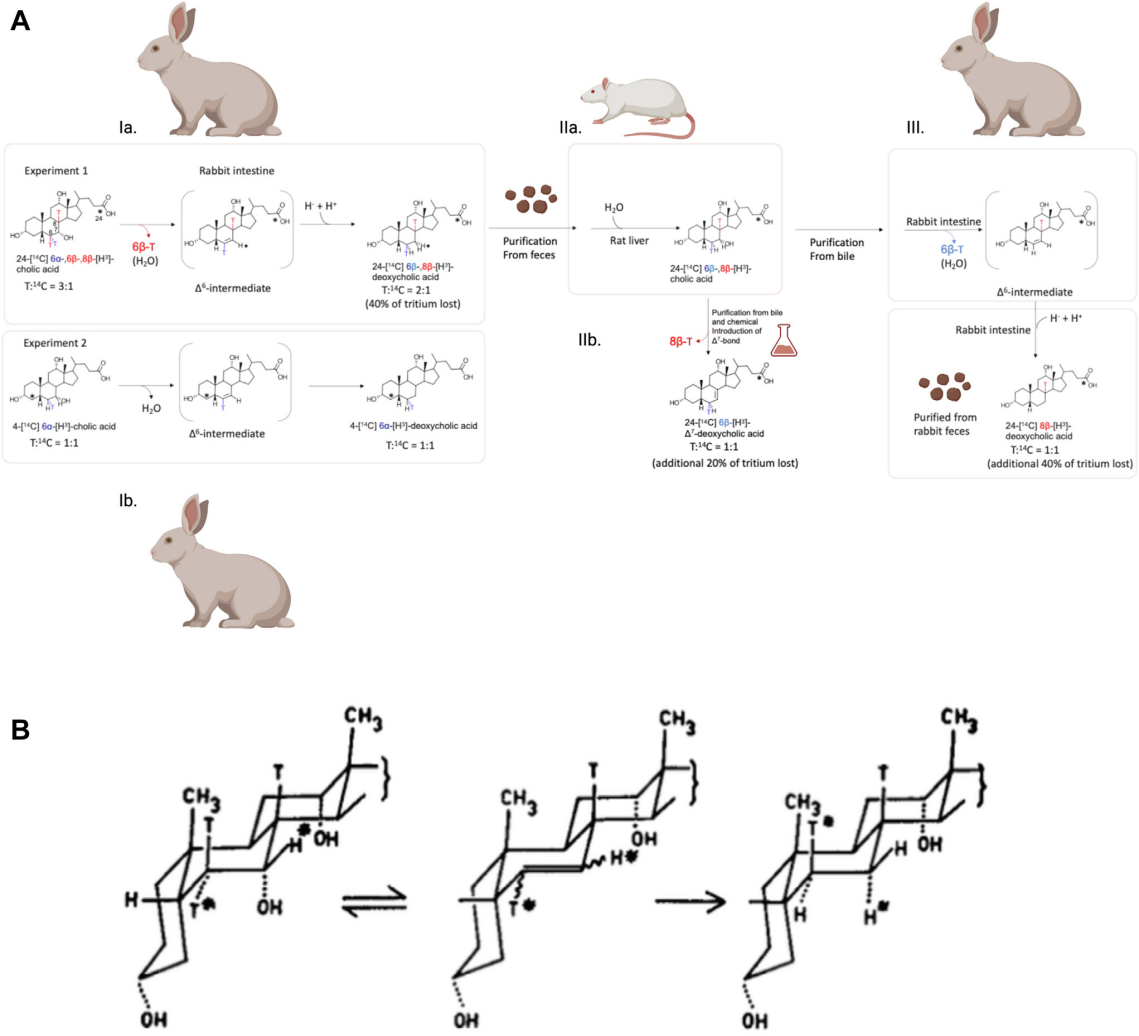
The Bergström-Samuelsson model of bile acid 7-dehydroxylation. A: A series of in vivo experiments administering [24-^14^C]6⍺-,6β-,8β-[^3^H]CA ([T]:[14C] ratio of 3:1 where T = tritium or ^3^H) (experiment 1) or [4-^14^C]6⍺-[^3^H]CA ([T]:[^14^C] ratio of 1:1) in rats and/or rabbits were performed in order to determine the stereochemistry for bile acid 7⍺-dehydroxylation. It was determined in previous studies that in [24-^14^C]6⍺-,6β-,8β-[^3^H]CA, 40% of the tritium was at 6⍺-position, 40% at 6β-position, and 20% at 8β-position (total 100%). In experiment 1, the radiolabeled CA was first administered to intact rabbits and DCA was recovered from feces, and the chromatographically purified compound was observed to have a [T]:[^14^C] ratio of 2:1 with a 40% loss of tritium (Ia.). From this experiment, it was clear that one of the C6-tritium labels was removed; however, it could not be determined which one from this experiment. Experiment 2 administered [4-^14^C]6⍺-[^3^H]CA to rabbits, and the chromatographically purified fecal DCA metabolite had a [T]:[^14^C] ratio of 1:1, indicating retention of the 6⍺-tritium (Ib.). Additional experiments were performed to further validate loss of the 6β-tritium and the fate of the 8β-tritium. The [24-^14^C]6β-,8β-[^3^H]DCA purified from rabbit feces in experiment 1 was then administered to bile duct cannulated rats (IIa.). Unlike the rabbit, the rat liver hydroxylates DCA, leading to removal of the 7⍺-[hydrogen] but retention of the 6β-tritium yielding [24-^14^C]6β-,8β-[^3^H]CA in bile. To determine the fate of the 8β-tritium, a Δ^7^-intermediate was chemically generated from the radiolabeled CA obtained from rat bile, resulting in a [T]:[^14^C] = 1:1 or another 20% loss of the tritium content (40% remaining), indicating elimination of the 8β-tritium (IIb.). To further confirm that the 6β-tritium is eliminated, the [24-^14^C]6β-,8β-[^3^H]CA obtained from rat bile was administered again to the rabbit and after fecal extraction, the [T]:[^14^C] ratio was reduced to 1:1 or another 40% loss of tritium (20% remaining) (III.). B: The original 1960 Samuelsson figure describing the proposed two-step mechanism of bile acid 7-dehydroxylation: (1) the 6β-tritium and 7⍺-OH is removed (diaxial trans-elimination); (2) the hydrogens added to C6 and C7 of the Δ6-intermediate are added trans to each other (inversion of the 7β-hydrogen to the 7⍺-position (denoted with star) and 6⍺-hydrogen is brought into the 6β-position), yielding DCA. Reprinted with permission (67). DCA, deoxycholic acid.

Following up on their observations, a subsequent model of bile acid 7-dehydroxylation was proposed in the 1960s by the Nobel laureates Bengt Samuelsson and Sune Bergström (66, 67, 68). The Samuelsson-Bergström model was based on elegant experiments determining the position and stereochemistry from which tritium loss occurred during the in vivo metabolism of [24-^14^C][6ɑ,6β,8β-^3^H]CA and [4-^14^C][6-^3^H]cholesterol (converted to [4-^14^C][6ɑ-^3^H]CA in the liver) in rats and rabbits (39, 69). The experimental results led to the proposal for a two-step mechanism (Fig. 4): a diaxial transelimination of the 7ɑ-hydroxyl group and 6β-hydrogen yielding 3ɑ,12ɑ-dihydroxy-5β-chol-6-enoic acid (**Δ**^6^-intermediate), followed by transhydrogenation of the **Δ**^6^-intermediate to DCA (39). Furthermore, the formation of a **Δ**^6^-intermediate is consistent with the observed retention of the C7-tritium in the “7-keto-intermediate” experiment (Fig. 3) (66, 67).

**Fig. 5:**
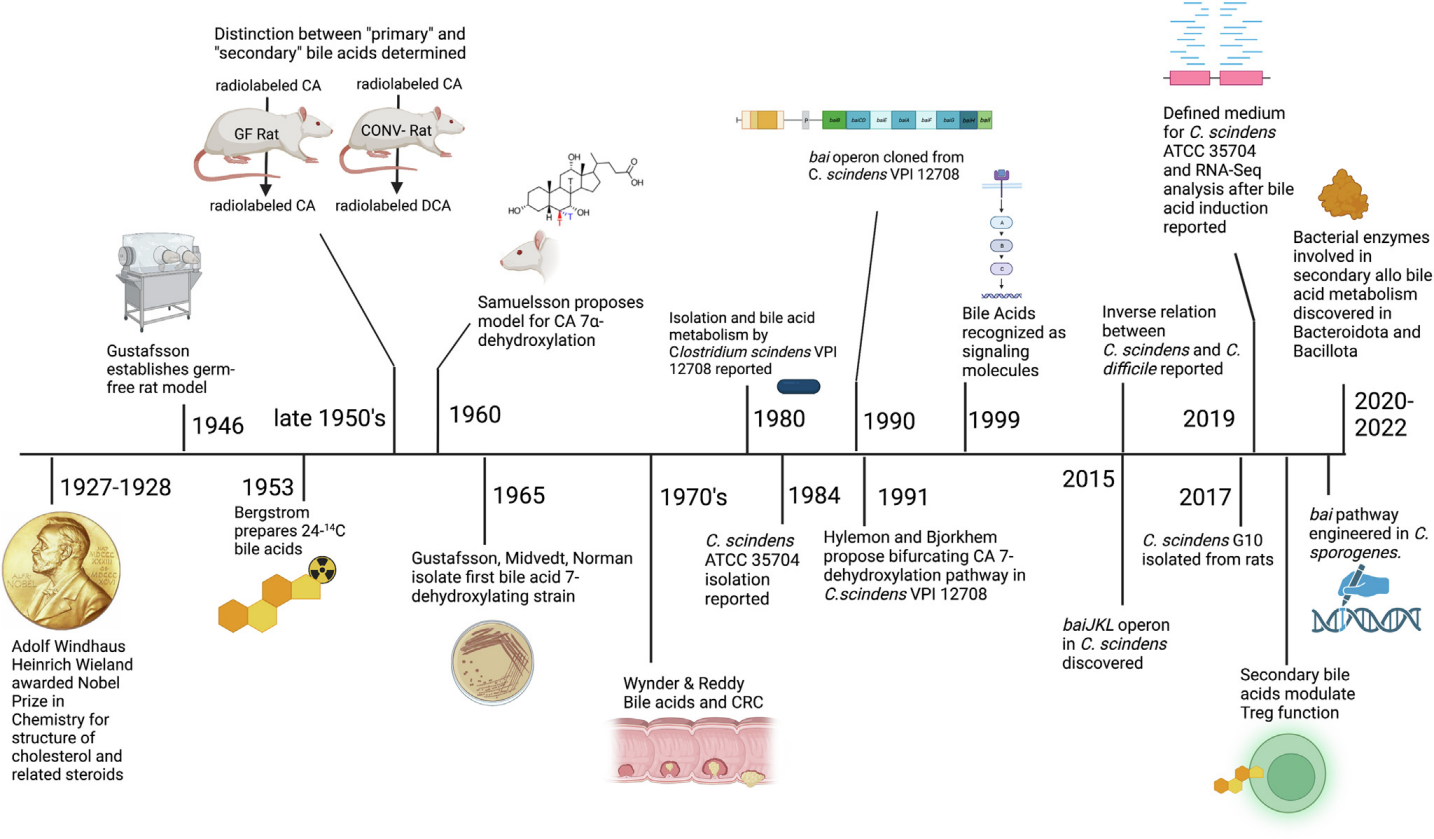
A timeline of key events in bile acid microbiology. (See text for descriptions).

The proposed **Δ**^6^-intermediate could not be identified in these early studies, due to the lack of bile acid 7-dehydroxylating bacterial isolates from which in vitro studies could be performed (39). However, support for this model came from chemical synthesis of the **Δ**^6^-intermediate, which when added to cultures of certain clostridia was converted to DCA (70, 71, 72, 73). Ironically, it was subsequent work in clostridia that revealed that the removal of the C7 hydroxyl group was far more complicated than initially thought (See Fig. 5 for Timeline).

## Isolation of bile acid 7-dehydroxylating bacteria

The first reported isolation of a bacterium capable of converting CA to DCA was at Harvard University in 1962, but the isolate was lost after a series of transfers in anaerobic medium (74). Successful isolation and maintenance of several bile acid 7-dehydroxylating bacterial isolates was reported in 1966 by Bengt Gustafsson, Tore Midtvedt, and Arne Norman at the Karolinska Institutet (75). The classification of their isolates, including “strain II”, were referred to as “lactobacillus” (75, 76), but the authors suggested these isolates “most nearly resemble the members of the genus *Clostridium*”. The inconclusive taxonomic assignment was due to uncertainty as to whether spores were observed, consistent with *Clostridium* or “sporelike” pleomorphisms characteristic of other genera (75).

Shortly thereafter, an isolate from rabbit feces suspected to be causal in the formation of gallstones composed largely of glycoallodeoxycholic acid was reported a few years later by Erwin Mosbach (Fig. 2) and associates at St. Luke’s Hospital in New York (77). A difficulty inherent in much of the history of steroid microbiology of the gut is that the fate of these strains is not clear; there is no record of deposition of these early strains in a culture collection. Moreover, it is always a task going back into earlier literature to determine the taxonomic methods used for identification since many of these have now changed. Early reports of the isolation of bile acid 7-dehydroxylating bacteria placed these organisms in the genera *Bacteroides* or *Lactobacillus* (68, 77, 78). Subsequent studies found no evidence that intestinal *Bacteroides* spp. convert CA to DCA (79), although recent work has revived the possibility of C7-dehydroxylation by *Bacteroides* strains (80). These characteristics, including lack of observable sporulation, are consistent with what has been learned about bile acid 7-dehydroxylating clostridia that have in recent decades been classified based on molecular phylogeny (81, 82, 83).

Most known bile acid 7-dehydroxylating isolates are in the *Clostridium* clusters and go by an increasingly expanding list of reclassified genera. Indeed, in 1969, Hattori and Hayakawa reported isolation of “*Bacteroides* strain 28S'' with bile acid 7ɑ-dehydroxylating activity (68). During the late 1970s, this strain was later reclassified as *Clostridium leptum* VPI 10900 by Holdeman and Moore at the “VPI Anaerobe Lab” at Virginia Tech (79, 84). Phillip Hylemon and coworkers demonstrated conversion of [24-^14^C]CA to [24-^14^C]DCA and [24-^14^C]CDCA to [24-^14^C]LCA by *C. leptum* VPI 10900 (79). Unfortunately, preparation of cell-free extracts from which to observe CA dehydroxylation intermediates could not be done with *C. leptum,* as activity was lost upon cell lysis even under strictly anaerobic conditions (79). It was around this time that *Eubacterium* sp. VPI 12708, later reclassified as *Clostridium scindens* VPI 12708 (81), was isolated from the stool of a colon cancer patient by Rainer Hammann in Bonn, Germany (85). Additional isolates were reported by Fusae Takamine (Fig. 2) (86), Seiju Hirano, Noriyuki Masuda and colleagues in Japan (87, 88), Thomas Clavel and colleagues in Germany (89, 90), and James E. Wells, Phillip B. Hylemon, and colleagues in the USA (91).

A landmark paper described the separation of bile acid 7-dehydroxylating activity into strains with “high” activity (*C. scindens and Peptacetobacter hiranonis*) and those with “low” activity (*Paeniclostridium sordelli, C. leptum, and Clostridium hylemonae*) based on ∼100-fold differences in conversion of CA to DCA (86). Starting with its initial characterization in 1980 at the VPI Anaerobe Lab (84), *C. scindens* VPI 12708 has served as a model organism (despite lack of genetic tractability) for resolution of the complex biochemistry and enzymology of bile acid 7-dehydroxylation (92). More recently, *C. scindens* ATCC 35074, isolated in the early 1980s by Victor Bokkenheuser (Fig. 2) and coworkers in New York, has been instrumental to the “omics” era in the analysis of bile acid 7-dehydroxylating bacteria in the human gut microbiome (93, 94, 95, 96).

## The Hylemon-Björkhem pathway of cholic acid 7-dehydroxylation

Another model for bile acid 7-dehydroxylation was developed decades after the work of Samuelsson and Bergström based on collaborations between Ingemar Björkhem and fellow steroid chemists at the Karolinska Institutet and Phillip Hylemon, a microbiologist at the Medical College of Virginia (now VCU School of Medicine) in the USA during the 1980s and 1990s.

**Fig. 6:**
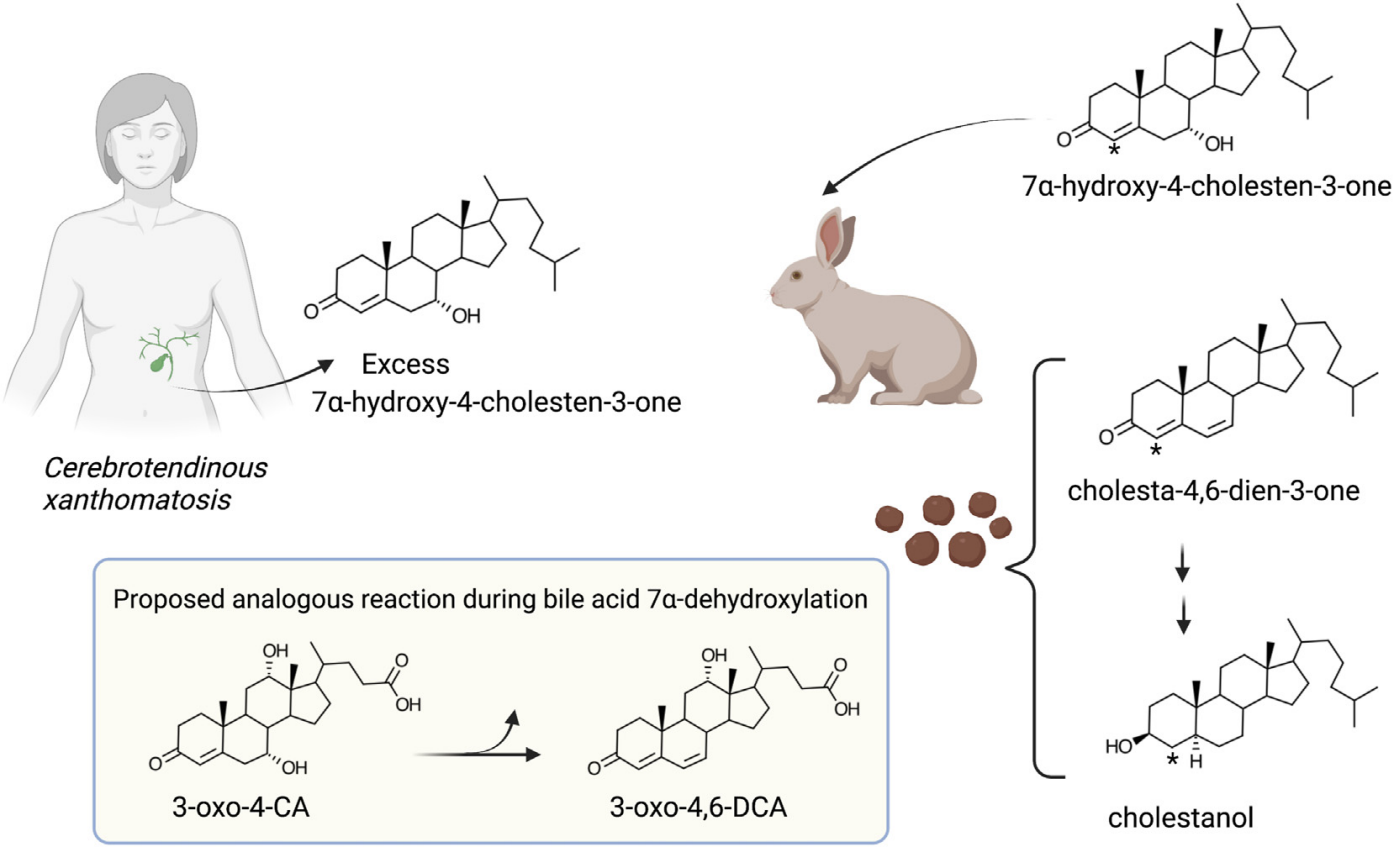
The metabolism of C4 in cerebrotendinous xanthomatosis patients and rabbits provided a potential mechanism for bile acid 7-dehydroxylation by intestinal bacteria. Studies by Ingemar Björkhem in CTX patients with radiolabeled 7⍺-hydroxy-4-cholesten-3-one (C4) indicated that the C7⍺-hydroxyl group was highly labile and converted to cholesta-4,6-dien-3-one and then to cholestanol by intestinal bacteria. An analogy was made between 7⍺-dehydroxylation of C4 and the 7⍺-dehydroxylation of CA and CDCA by intestinal bacteria, in a mechanism distinct from that proposed in the Bergström-Samuelsson model, which did not include a 3-oxo-**Δ**^4^-steroid intermediate. CDCA, chenodeoxycholic acid; CTX, cerebrotendinous xanthomatosis.

The Björkhem lab focused studies on the formation of cholestanol (5α-cholestan-3β-ol) in patients with the rare inborn error in bile acid metabolism called cerebrotendinous xanthomatosis (CTX) (97). Aside from identifying 7α-hydroxy-4-cholesten-3-one (C4) as a biological marker for bile acid synthesis in the liver (98), the Björkhem lab determined that patients with CTX had a marked increase in biliary excretion of C4 (97). It was hypothesized that C4 was a key intermediate in the formation of cholestanol, which accumulates in xanthomas in CTX patients. Skrede and Björkhem (1982) observed that [4-^14^C]C4 was converted by the intestinal microbiota of rabbits to [4-^14^C]cholesta-4,6-dien-3-one and 4-cholesten-3-one (99). In this work, the authors drew the analogy between a possible mechanism of bile acid 7α-dehydroxylation by *C. scindens* VPI 12708 and the 7α-dehydroxylation of C4 by intestinal bacteria (Fig. 6) (99) and later in liver microsomes (100). This novel hypothesis suggested the formation of a 3-oxo-**Δ**^4^-bile acid intermediate and C7-dehydration, yielding a 3-oxo-**Δ**^4,6^-intermediate not predicted by the Samuelsson-Bergström model nor from the then contemporary work in *C. scindens* VPI 12708.

The formation of 3-oxo-**Δ**^4^-CA proceeds through oxidation of the 3ɑ-hydroxy group (removal of the 3ɑ-hydroxy proton and 3β-hydride) followed by introduction of a double bond in the A ring (C4-C5 dehydrogenation). The administration of CA tritiated at C3 or C5 as well as coadministration of [24-^14^C]CA to healthy human volunteers followed by collection of duodenal bile provided a key in vivo test of both the Samuelsson-Bergström model and the mechanism proposed by Björkhem (101, 102). The same experiments were performed with whole cells or cell extracts of *C. scindens* VPI 12708. In both in vivo and in vitro experiments, the tritium label was removed during CA conversion to DCA giving strong support for the “3-oxo- **Δ**^4^-CA intermediate” hypothesis which was analogous to C4 predicted by Skrede and Björkhem. In this scheme, C7-dehydroxylation would then yield a “Δ^6^-intermediate” (3-oxo-**Δ**^4,6^-DCA) distinct from the “Δ^6^-intermediate” (3ɑ,12ɑ-dihydro-5β-chol-6-enoic acid) predicted by Samuelsson and Bergström (Fig. 4).

**Fig. 7:**
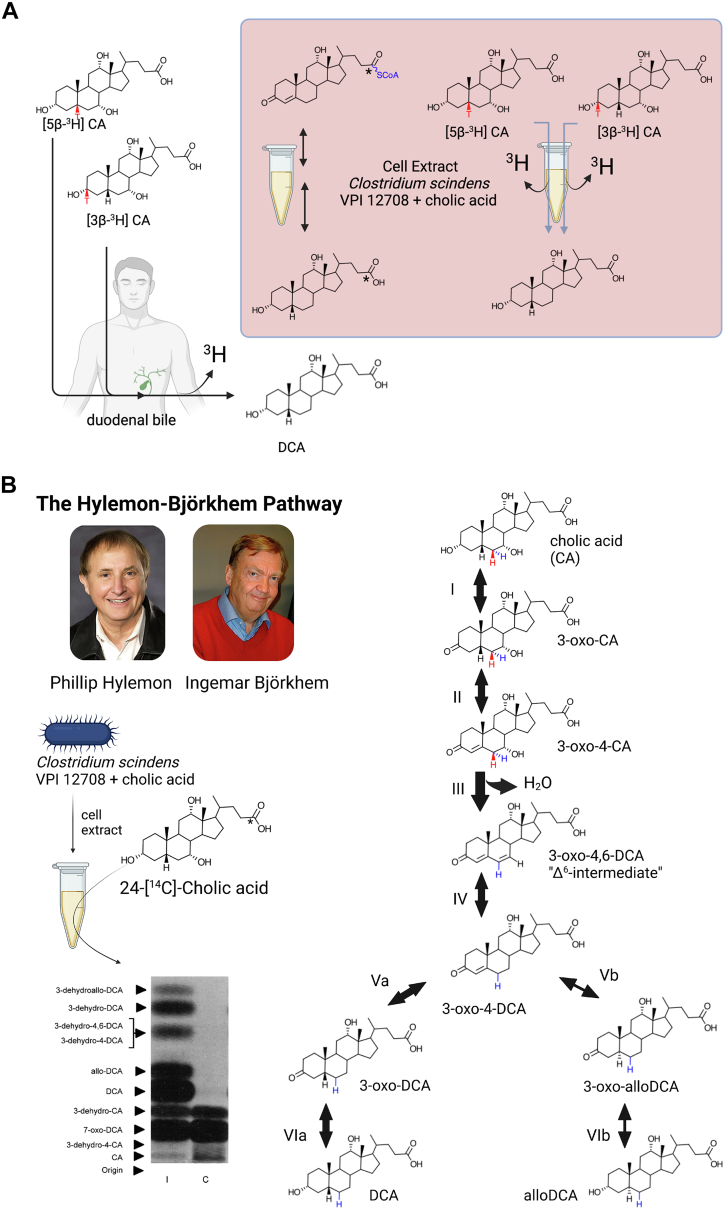
The Hylemon-Björkhem pathway of cholic acid 7-dehydroxylation. A: To determine whether a 3-oxo-**Δ**^4^ intermediate was involved in bacterial 7⍺-dehydroxylation of bile acids, Hylemon and Björkhem determined the fate of [3β-^3^H]CA + [24-^14^C]CA and [5β-^3^H]CA + [24-^14^C]CA in human volunteers and cell extracts of *Clostridium scindens* VPI 12708, a model bile acid 7⍺-dehydroxylating human gut bacterium. These experiments confirmed the loss of [3β-^3^H] and [5β-^3^H] labels during conversion of CA to DCA indicating formation of a 3-oxo-**Δ**^4^ intermediate inconsistent with the Bergström-Samuelsson model. B: Further studies by Phillip B. Hylemon at VCU Medical School and Ingemar Björkhem at the Karolinska Institutet (pictured) with [24-^14^C]CA in cell extracts of *C. scindens* VPI 12708 yielded a complex pattern of CA intermediates when cells were induced by CA (“I”) relative to cells grown without bile acids (“C”). Each bile acid intermediate was scraped from the TLC plate, and structures were determined by MS/MS analysis. Each bile acid intermediate was chemically synthesized and shown to be converted to DCA in cell extracts of *C. scindens* VPI 12708, verifying these as intermediates in the pathway (except for 7-oxoDCA). Unexpectedly, derivatives of allodeoxycholic acid (alloDCA) were also detected. Identification of these CA intermediates led to the elucidation of the complex, bifurcating Hylemon-Björkhem pathway of cholic acid 7-dehydroxylation. We have highlighted the hydrogens (red & blue) that Samuelsson had tritiated at carbon-6 for reference. DCA, deoxycholic acid.

The above results are also consistent with the formation of 3-oxo-**Δ**^4^-DCA, one of the reductive products on the path from 3-oxo-**Δ**^4,6^-DCA to DCA, observed in the cell extracts of *C. scindens* VPI 12708 (102). Indeed, the Hylemon lab subsequently observed the formation of hydrophilic, potentially conjugated CA intermediates during the incubation of radiolabeled unconjugated bile acids with cell-free extracts of *C. scindens* VPI 12708 (99). After addition of [24-^14^C]DCA to cell extracts, they identified conjugation to a nucleotide analog that would later be identified as coenzyme A (99). In collaboration with Jan Svöjall, the bile acid linked to CoA in the cell extracts of *C. scindens* VPI 12708 was determined to be 3-oxo-**Δ**^4^-DCA. This indicated that cell extracts were catalyzing in the oxidative direction from DCA → 3-oxo-DCA → 3-oxo-**Δ**^4^-DCA (Fig. 7A). In earlier studies by Kallner, radiolabeled 3-oxo-**Δ**^4^-DCA was injected into the reductive environment of the rat cecum where it was converted primarily to radiolabeled DCA (103, 104). While tempting to interpret the in vivo studies of Kallner as early support for 3-oxo-**Δ**^4^-DCA as an intermediate in bile acid 7-dehydroxylation, other bacteria, such as *Clostridium paraputrificum* and *Bacteroides* sp., lack the ability to convert CA to DCA but express enzymes that reduce 3-oxo-**Δ**^4^-steroids (105, 106, 107).

Analyzing the multiple products formed after incubation of radiolabeled CA with cell extracts of *C. scindens* VPI 12708 led Hylemon and Björkhem to propose a detailed biochemical pathway for CA conversion to DCA by intestinal clostridia (Fig. 7B). Addition of each chemically synthesized, radiolabeled CA intermediate to cell extracts of *C. scindens* VPI 12708 resulted in DCA formation, consistent with the hypothesized mechanism (108). We therefore propose that the pathway deduced from their studies be named the Hylemon-Björkhem pathway (Fig. 7B).

## Bai genes

**Fig. 8:**
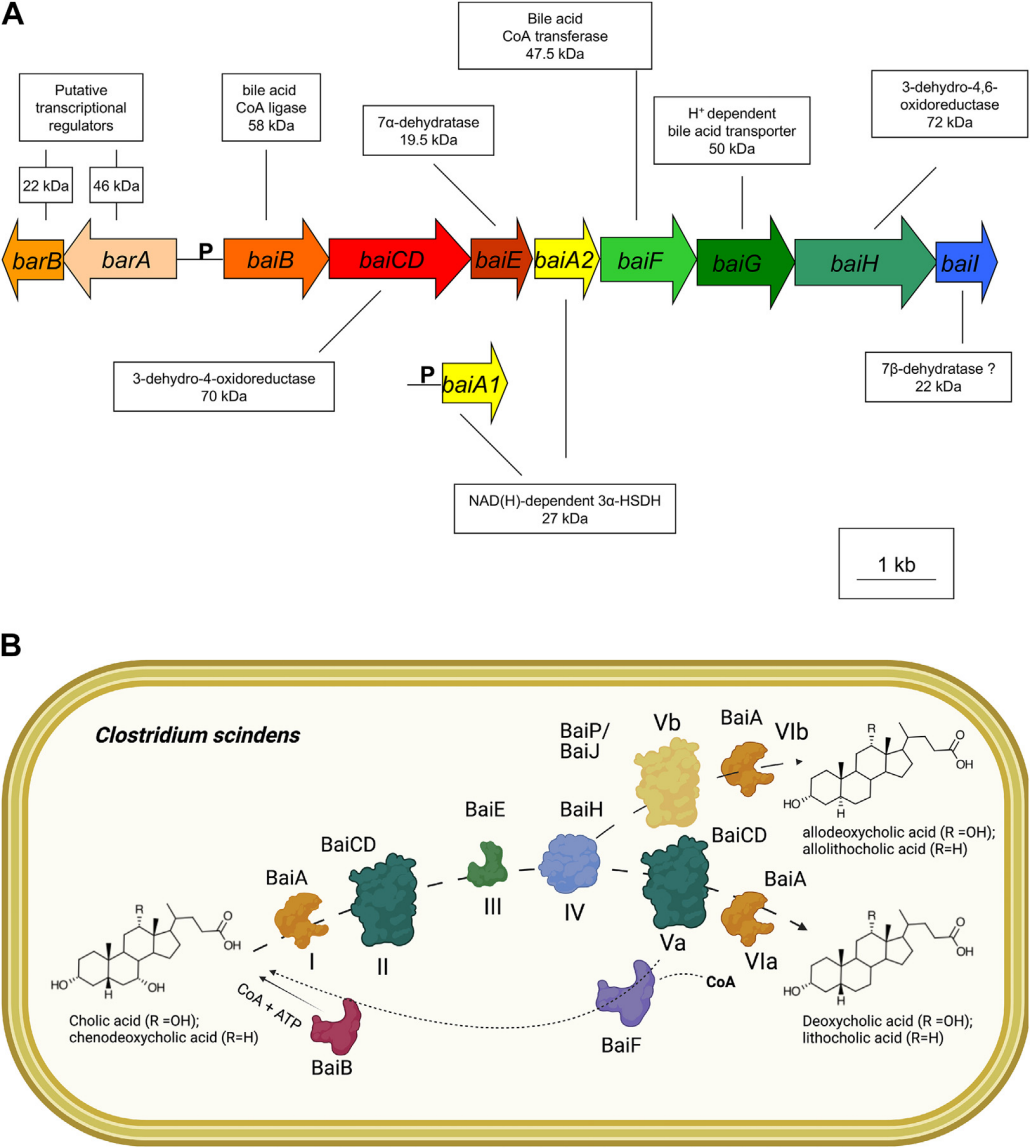
The bile acid inducible operon and bile acid enzymes catalyzing each step of the Hylemon-Björkhem pathway. A: The bile acid inducible (bai) regulon in *Clostridium scindens* VPI 12708 has been identified (Modified from Ridlon *et al.* (92)). B: Enzymes corresponding to each reaction step of the Hylemon-Björkhem pathway (I-VIa/VIb), as well as enzymes involved in bile acid-coenzyme A metabolism.

The identification and characterization of Bai enzymes over the ensuing three decades, mainly in the Hylemon lab, further support the Hylemon-Björkhem pathway of CA 7-dehydroxylation. Early studies determined that the expression of Bai proteins was inducible by the addition of CA or CDCA, but not the 7β-hydroxy epimer of CDCA, ursodeoxycholic acid (UDCA), or the secondary bile acid, DCA (85, 109). Comparison of two-dimensional gels from control and CA-induced whole cells of *C. scindens* VPI 12708 led to identification of several inducible polypeptides (71). N-terminal sequencing of CA-inducible polypeptides led to a reverse genetic approach to clone the ∼12-kb *bai* operon in its entirety a decade later by the Hylemon lab (110). The order of *bai* genes is largely conserved among intestinal clostridia, and in *C. scindens* VPI 12708 strain, it is *baiBCDEAFGHI* (Fig. 8A) (111).

Deconjugation of bile acids is a prerequisite for bile acid 7-dehydroxylation (79, 109, 112, 113). The inhibition of bile salt hydrolase (BSH) activity should thus also decrease the rate of bile acid 7-dehydroxylation, as has recently been demonstrated (114, 115). Studies reported in the 1980s on cofactor requirements for DCA formation with dialyzed cell extracts of *C. scindens* VPI 12708 indicated that the NAD^+^/NADH ratio and addition of flavins are important for activity (71, 72, 85). Molecular cloning and much of the enzymology of the Hylemon-Björkhem pathway were worked out in the Hylemon lab (92, 111). Unconjugated primary bile acids are imported into the cell by a 50-kDa proton-dependent transporter encoded by the *baiG* gene (116). Consistent with the identification of coenzyme A conjugates in the cell extracts of *C. scindens* VPI 12708, free bile acids were shown to be ligated to coenzyme A in an ATP-dependent manner through the bile acid CoA ligase (BaiB) or through coenzyme A transferase activity (BaiF, BaiK) (117, 118, 119). The conversion of CA to DCA in cell extracts of *C. scindens* VPI 12708 is enhanced by the addition of NAD^+^, a cofactor important in oxidoreduction reactions (72). Bile acids with a 3ɑ-hydroxy group (CA, CDCA, and UDCA) or 3-oxo-group (3-oxo-CA, 3-oxo-CDCA, 3-oxo-UDCA) are substrates for bile acid 7-dehydroxylation, while iso-bile acids (3β-hydroxy) are not substrates (120). This is because the first oxidation step is catalyzed by NAD^+^-dependent 3ɑ-HSDH encoded by the *baiA1* and *baiA2* genes (121, 122). The BaiA1 and BaiA2 enzymes are unique among HSDH enzymes in being specific for coenzyme A conjugates (121, 122). The rational formation of iso-CA and iso-CDCA may represent a strategy to decrease the rate of formation of secondary bile acids such as DCA and LCA. After formation of 3-oxo-cholyl∼SCoA, the 72-kDa flavoprotein, BaiCD, catalyzes a stereospecific NADH-dependent oxidation yielding 3-oxo-**Δ**^4^-cholenyl∼SCoA (123). Taken together, CoA addition followed by oxidations catalyzed by BaiA and BaiCD yields the “3-oxo-**Δ**^4^-intermediate” identified by Hylemon and Björkhem.

The Hylemon lab originally chromatographically separated “7ɑ-dehydroxylase” and “NADH:flavin oxidoreductase” activities from cell extracts of *C. scindens* VPI 12708. They later determined that the rate-limiting 7ɑ-dehydration catalyzed by the 19.5-kDa BaiE reaction results in the formation of chemically stable conjugated double-bond system in the 3-oxo-**Δ**^4,6^-DCA∼CoA intermediate (124). Work with analogous structures (i.e., C4) indicates that dehydration proceeds by both an enzymatic mechanism, as well as spontaneous dehydration through raising the pH into the basic range (99, 100). There is some uncertainty as to whether this reaction involves a *trans* elimination or *cis* elimination of water, with the former suggested by radiolabel studies, while the latter is suggested by X-ray crystallography (39, 125). It is also unclear at what point the majority of CoA is hydrolyzed or transferred. The BaiE recognizes both 3-oxo-**Δ**^4^-cholenyl∼SCoA and 3-oxo-**Δ**^4^-cholenoic acid (125). A 3-oxo-**Δ**^4^-deoxycholenyl∼CoA intermediate has also been observed in cell extracts, suggesting CoA transfer may occur after dehydration (102).

The “NADH:FOR” protein was determined to be the flavin-dependent BaiH (126, 127), which catalyzes the formation of 3-oxo-**Δ**^4^-UDCA from 3-oxoUDCA (123). Recent work has identified the BaiH as also catalyzing bile acid **Δ**^6^-reductase activity, converting 3-oxo-**Δ**^4,6^-DCA to 3-oxo-**Δ**^4^-DCA (128). It is thus highly probable that the conversion of Samuelsson’s **Δ**^6^-intermediate (3ɑ,12ɑ-dihydroxy-5β-chol-6-enoic acid) to DCA in cell extracts of *C. scindens* VPI 12708 was catalyzed by the BaiH or BaiN (129), although this has not yet been demonstrated. The BaiCD was shown to reduce 3-oxo-**Δ**^4^-DCA to 3-oxo-DCA (128). Finally, the BaiA1 and BaiA2 catalyze the final reductive step, yielding DCA (121). Candidate efflux transporters involved in the export of secondary bile acids have recently been identified through transcriptomic analysis after CA induction of *C. scindens* ATCC 35704 (94). Engineering of the *baiBACDEFH* genes into *Clostridium sporogenes* in the Fischbach lab has been shown to be sufficient to confer biochemical conversion of CA to DCA to this nonbile acid 7-dehydroxylating species (128). In vitro incubation of BaiB, BaiA, BaiCD, BaiE, BaiF, and BaiH with ATP, CoA, and NAD^+^ was both necessary and sufficient to convert CA to DCA (128). Recent work also demonstrates that the *baiN* gene product functions in an analogous two-step reaction catalyzed by BaiH and BaiCD (129). Taken together, Bai enzymes catalyzing each step of the Hylemon-Björkhem pathway from CA to DCA have been identified (Fig. 8B).

## A short digression on early hints of the mechanism of CA degradation

The microbiologist Stanley Falkow famously stated that, “The world is covered in a fine patina of feces” (130). Our agricultural efforts bear this out. The manuring of soils creates rich sources of carbon, including bile salts. A gram of animal manure contains roughly 1–8 mg of bile salts, and manured soil concentrations of bile acids are estimated in the millimolar range. It is thus not surprising that soil microbiota have evolved complex biochemical pathways to mineralize bile salts to CO_2_ (131).

Evidence for a multistep C7 dehydroxylation of CA by the aerobic soil bacterium *Arthrobacter simplex* was reported by Hayakawa and Samuelsson (1964) at the Karolinska Institutet (132). Rigorous identification of bile acid intermediates included 3-oxoCA, 3-oxo-**Δ**^4^-CA, 3-oxo-**Δ**^1,4^-DCA, 3-oxo-**Δ**^4,6^-DCA, and 3-oxoDCA (132). In the introduction of the Hayakawa and Samuelsson paper, the authors lamented the barriers to studying the analogous pathway in anaerobic bacteria in human stool samples: “The predominant microbiological reaction involves elimination of the 7ɑ-hydroxyl group of cholic acid and chenodeoxycholic acid, leading to formation of deoxycholic acid and lithocholic acid. This reaction has been demonstrated in vitro with microorganisms obtained from rat and man. However, despite considerable efforts directed at the isolation of the microorganism(s) responsible for this transformation, it has so far not been possible to identify the microorganism(s) involved. For this reason, the mechanism of the reaction has only been studied in vivo with bile acids specifically labeled with tritium. However, nothing has been learned about the enzymatic details.”(132).

Two years later, in 1966, pure cultures of seven human fecal bacteria capable of bile acid 7-dehydroxylation were isolated at the Karolinska Institutet by Gustafsson, Midtvedt, and Norman. Thus, Samuelsson possessed the methodology for identifying the key 3-oxo-**Δ**^4^-DCA and 3-oxo-**Δ**^4,6^-DCA intermediates in aerobic bile acid degradation in soils and for confirming the reduction of the C6-C7 double bond during enterohepatic circulation of radiolabeled CA in humans, and shortly thereafter, in C7-dehydroxylating stool bacteria that had been isolated by collaborators at the same University (75). In hindsight, the ingredients were there for bile acid pathway intermediates to have been outlined decades earlier. However, the research focus of Bergström and Samuelsson had shifted towards prostaglandins during the early 1960s once their group moved from Lund University to the Karolinska Institutet, work for which they would later share the Nobel Prize in Physiology and Medicine (133).

## Bacterial shape-shifting of bile acids

**Fig. 9:**
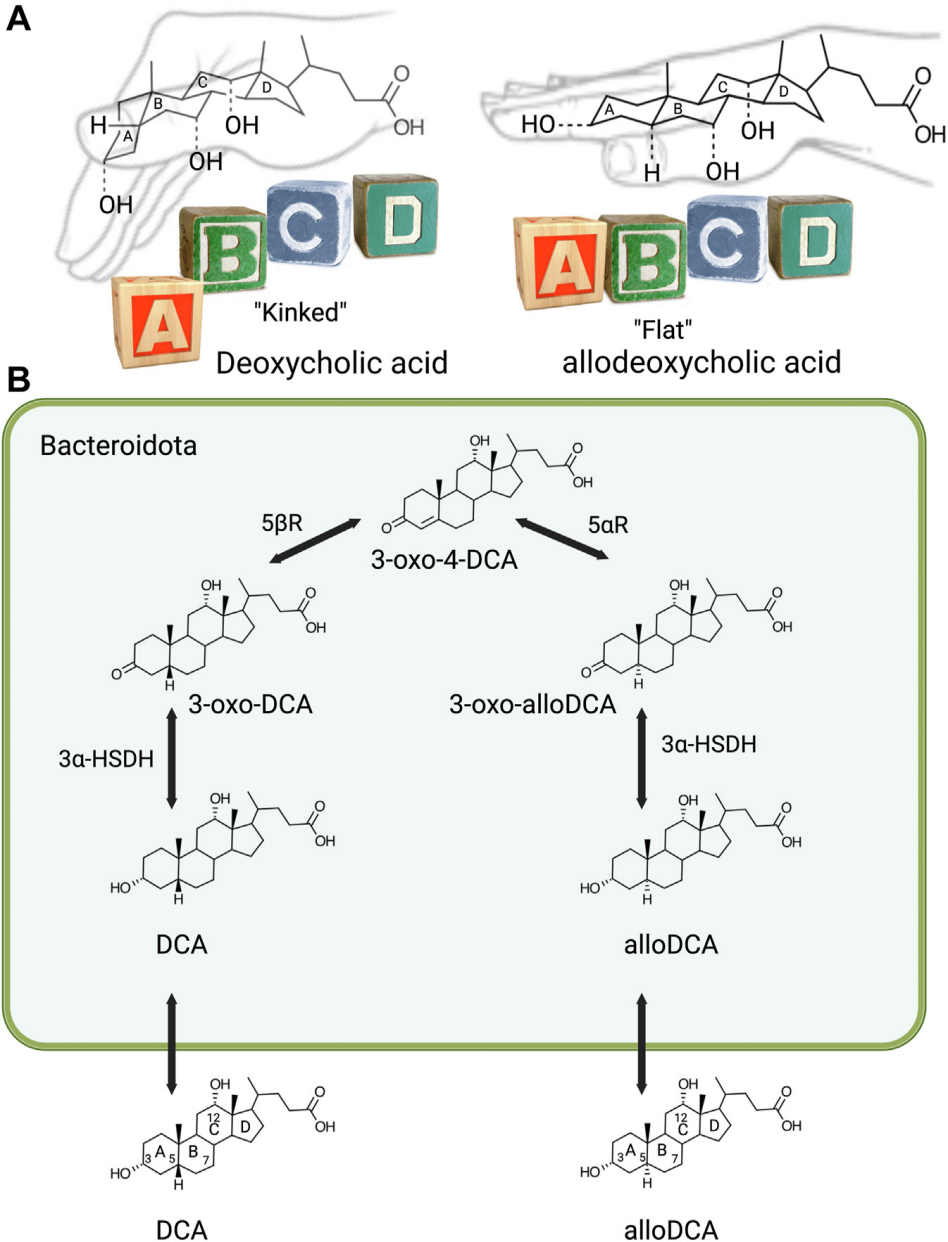
An indirect pathway for the microbial formation of allo-secondary bile acids. A: Allo-bile acids are “flat” owing to the A/B-trans ring structures, while host bile acids and secondary bile acids are “kinked” due to A/B-cis ring structures. B: The pathway from CA to alloDCA can be thought of as the “direct pathway”, but an “indirect pathway” to alloDCA catalyzed by enzymes encoded by the *Bacteroidota* results in an equilibrium between DCA and alloDCA through a 3-oxo-**Δ**^4^-DCA intermediate. So far, the *Bacteroidota* have not been shown to encode a complete set of bai-like genes, instead, expression of 3⍺-HSDH, 3-ketosteroid-5β-reductase (5βR), and 3-ketosteroid-5⍺-reductase (5⍺R) allow isomerization of the A/B-rings. alloDCA, allodeoxycholic acid; Bai, bile acid-inducible; DCA, deoxycholic acid; HSDH, hydroxysteroid dehydrogenase.

The usefulness of a scientific model is determined by its ability to incorporate and explain new phenomena. The “3-oxo-**Δ**^4^-intermediate” introduced by the Hylemon-Björkhem pathway raises the possibility for the formation of secondary bile acid isomers with distinct steroid ring shapes not predicted by the Samuelsson-Bergström model. So far, we have described the conversion of “kinked” primary bile acids produced by the human liver to “kinked” secondary products such as DCA and LCA. However, microbes can generate “flat” stereoisomers known as secondary “allo” bile acids which include allodeoxycholic acid (alloDCA) and allolithocholic acid (alloLCA) (Fig. 9A) (103, 104). AlloDCA was first isolated from rabbit bile by Kishi in 1936, who misidentified it as “lagodeoxycholic acid” (the 12β-hydroxy epimer of DCA) but was properly identified decades later after purification from rabbit feces and bile by Danielsson and colleagues (1963) (134) and from rabbit gallstones by Alan Hofmann and Erwin Mosbach (both pictured in Fig. 2) (135). Indeed, early studies reported the formation of gallstones enriched with glycoallodeoxycholic acid in rabbits fed 5ɑ-cholestan-3β-ol (135, 136). Gallstone formation in this model was prevented through neomycin treatment (137), and a neomycin-sensitive fecal isolate “FA 1/146” that converted CA to DCA and alloCA to alloDCA isolated by Bokkenheuser, Mosbach, and colleagues indicated a causal role for the gut microbiome in generating alloDCA, which is absorbed and accumulates in bile in this model (77).

In 1991, Hylemon *et al.* (108) reported the formation of [24-^14^C]alloDCA as a product of [24-^14^C]CA metabolism in cell extracts of *C. scindens* VPI 12708. This indicates that the Hylemon-Björkhem model is a multistep, bifurcating pathway that can only be explained through formation of 3-oxo-4 intermediates (Fig. 7B) (108). In the path from CA (5β-H) to DCA (5β-H) via a 3-oxo-**Δ**^4^ intermediate, the A/B-ring stereochemistry is retained. The BaiCD is a 3-keto-Δ^4^-5β-oxidoreductase enzyme responsible for removal and reintroduction of the 5β-H during 7-dehydroxylation of CA (Figs. 7B and 8B) (128). However, an alternative path from CA (5β-H) to alloDCA (5ɑ-H), which changes the A/B ring stereochemistry, leads to the prediction that *C. scindens* encodes a bile acid 3-keto-Δ^4^-5ɑ-oxidoreductase enzyme that utilizes 3-oxo-**Δ**^4^-DCA and 3-oxo-**Δ**^4^-LCA as substrates.

## Enzymatic basis for allo-secondary bile acid formation in the Hylemon-Björkhem pathway

In 2019, the complete genome and global transcriptomic responses of *C. scindens* ATCC 35704 to bile acids was reported (94). Among genes significantly upregulated by CA induction was a predicted pyridine nucleotide-dependent urocanate reductase that we hypothesized to encode a bile acid 5ɑ-reductase involved in the formation of alloDCA (94). Our subsequent work confirmed that this gene, named *baiP*, encodes a bile acid 5ɑ-reductase which acts in concert with BaiA1 to convert 3-oxo-**Δ**^4^-DCA to alloDCA and 3-oxo-**Δ**^4^-LCA to alloLCA (138). Prior work by Ridlon and Hylemon identified a *bai* gene polycistron that was named *baiJKL* and was located adjacent to *baiA*1 in *C. scindens* VPI 12708 and *C. hylemonae* TN271 (117). The BaiK is a homolog of the BaiF which is involved in bile acid CoA transfer and shares this function (117). The *baiJ* was predicted to encode a flavin-dependent 3-ketosteroid dehydrogenase hypothesized to be involved in sterol A/B ring oxidoreduction. Phylogenetic analysis of BaiP from *C. scindens* ATCC 35704 revealed BaiJ as a homolog. Functional characterization of recombinant BaiJ from *C. scindens* VPI 12708 expressed in *E. coli* confirmed the conversion of 3-oxo-**Δ**^4^-DCA and 3-oxo-**Δ**^4^-LCA to allo-secondary bile acids (138). Taken together, the identification of *baiJ* and *baiP* genes supports the observed bifurcation proposed by the Hylemon-Björkhem pathway, yielding both DCA and alloDCA, through a 3-oxo-**Δ**^4^-DCA intermediate.

Phylogenetic analysis and Hidden Markov model searches of BaiJ, BaiP, and BaiE, and BaiCD in human gut metagenomes revealed enrichment in colorectal cancer patients relative to adenoma and healthy controls (138). The *Oscillospiraceae* was recently identified as a novel group of uncultured bile acid 7-dehydroxylating bacteria (139), and these taxa were also identified as having the genetic potential for generating secondary allo-bile acids (138). We have referred to the formation of secondary allo-bile acids from primary bile acids as the “direct pathway” since primary bile acids are converted to secondary allo-bile acids by a single bacterial strain (138). There are other mechanisms by which secondary allo-bile acids can be generated by gut microbiota through the metabolism of DCA and LCA.

## An “indirect pathway” to secondary allo-bile acids

The indirect pathway describes the equilibrium between DCA and alloDCA or LCA and alloLCA through a 3-oxo-**Δ**^4^-intermediate (Fig. 9B). This “indirect pathway” first requires conversion of CA to DCA or CDCA/UDCA to LCA by one bacterial strain, followed by oxidation and ring epimerization by another strain. The in vivo conversion of radiolabeled DCA to radiolabeled alloDCA after intracecal injection in rabbits was first reported in 1963 by Danielsson (134). A biochemical pathway was proposed in 1967 by Anders Kallner, who studied the metabolic fate of tritiated DCA, 3-oxo-DCA, 3-oxo-**Δ**^4^-DCA, 3-oxo-alloDCA, and alloDCA by the gut microbiota in rats (103, 104). In all cases, a mixture of radiolabeled DCA and alloDCA was obtained, confirming an equilibrium between these compounds in the GI tract (Fig. 9B). Loss of the 3β-tritium from [3β-^3^H][24-^14^C]DCA during in vivo conversion to [24-^14^C]alloDCA confirmed the formation of a 3-oxo intermediate (103, 104).

A later study by Edenharder (1988) reported the conversion of 5β-bile acids to 5ɑ-bile acids by fecal *Bacteroides* isolates (78). Work in the chicken microbiome identified a gene annotated as “steroid 5ɑ-reductase” (5AR) similar to mammalian *SRD5A1* widespread among the *Bacteroidota* (140). A recent study (107) by Kenya Honda and colleagues reported that strains of *Parabacteroides merdae*, *Odoribacter laneus,* and *Bacteriodes dorei* incubated with 3-oxo-**Δ**^4^-LCA resulted in conversion to isoalloLCA. This confirms that the *Bacteroidota* (formerly *Bacteroidetes*) express 5AR that recognize bile acids and indicate the expression of bile acid 3β-HSDH (described in the next section) (107). Twenty other *Bacteroidota* strains were shown to convert 3-oxo-**Δ**^4^-LCA to 3-oxo-alloLCA, and disruption of the 5AR gene in *P. merdae* prevented the formation of alloLCA derivatives (107). The 5AR gene was clustered with predicted 3β-HSDH and NADH:flavin oxidoreductase (similar to BaiCD and BaiH), hypothesized to function as bile acid 5β-reductase. Several strains were found to convert secondary bile acids such as 3-oxoLCA or isoLCA (5β-reduced) to isoalloLCA. However, the conversion of CDCA to isoalloLCA required the combined metabolism of *C. scindens* (capable of C7-dehydroxylation) with *Bacteroidota* strains (107). So far, the *Bacteroidota* have not been shown to encode a complete set of *bai*-like genes. The contribution of *C. scindens* to allo-secondary bile acid production (through the direct pathway) in coculture with *Bacteroidota* strains is not clear. Collectively, these data demonstrate that *Bacteroidota* strains possess an “indirect pathway” allowing isomerization of the bile acid A/B-rings.

While isoalloLCA derivatives in stool are measured at relatively low micromolar concentrations, these concentrations (1–3 μM isoalloLCA) are sufficient to inhibit the growth of gram-positive pathogens, including *Clostridioides difficile* (107). Thus, while “kinked” secondary bile acids DCA and LCA are inhibitory against many gram-negatives (141), the formation of “flat”, 3β-derivatives such as isoalloLCA appear to be inhibitory for many gram-positive bacteria (86). The relative contributions of the direct and indirect pathways to the formation of secondary allo-bile acids in the GI tract are unexplored.

## The special case of bile acids with an epimerized C7 hydroxyl group

Navigators and watchers of the night sky, both professional and amateur, look to the handle of the constellation Ursa Minor (“little bear”) to find the North Star. For bile acid researchers, “urso” bile acids hold a special affection. UDCA is a major primary biliary bile acid of bears (genus *Ursus*) (22, 142) and has been utilized in traditional medicine, along with many other animal biles, for centuries (22). There is a “little bear” in all of us, as judged by the measurement of minor quantities of UDCA in feces, blood, and bile (21). Unlike bears (and mice), whose “urso” biliary component is produced primarily by enzymes encoded in their genome (thus a primary bile acid), it is the epimerization action of the human gut microbiota responsible for the formation of UDCA as a secondary bile acids in humans (21). UDCA is synthesized on the order of 1,000 metric tons for therapeutic uses each year, primarily for the treatment of primary biliary cholangitis (21).

The Hylemon-Björkhem model in humans is further complicated in the case of 7β-hydroxy bile acids such as the therapeutic bile acid, UDCA. Two mechanisms have been determined for the removal of the 7β-hydroxy group from UDCA: *1*) epimerization of UDCA to CDCA followed by 7ɑ-dehydroxylation by the concerted action of intestinal bacteria and *2*) direct 7β-dehydroxylation by species such as *C. scindens* (143).

Regarding the second mechanism, it is presumed, although not yet demonstrated, that the BaiG imports UDCA which is then ligated to CoA by the BaiB, BaiF, or BaiK, followed by oxidation by BaiA (143). Prior work provided qualitative evidence that the BaiH is a stereospecific enzyme involved in the second oxidative step involved in the formation of 3-oxo-**Δ**^4^-UDCA(∼SCoA), while the BaiCD catalyzes the same step with 7ɑ-hydroxy bile acid epimers (123). The BaiE is stereospecific and does not recognize 3-oxo-**Δ**^4^-UDCA∼SCoA as a substrate. Indeed, bile acid 7ɑ-dehydratase (BaiE) and 7β-dehydratase activity in *C. scindens* can be detected and separated by FPLC (124). The *bai*I gene product is a homolog of BaiE in the SnoAL_4 family of proteins, the only gene in the *bai* operon for which a function has yet to be ascribed and is hypothesized to have bile acid 7β-dehydratase activity (92).

After the rate-limiting 7β-dehydratase step, a 3-oxo-**Δ**^4,6^-LCA derivative is formed as is also the case with the 7ɑ-dehydration of CDCA. Thus, reduction of this intermediate is expected to follow the aforementioned BaiH → BaiCD→ BaiA path towards LCA or BaiH→BaiP/BaiJ→BaiA path towards alloLCA. Thus, 7ɑ-dehydroxylation and 7β-dehydroxylation are predicted to proceed by the same series of steps, with the substitution of stereospecific enzymes at the step leading up to and including 7β-dehydration.

## Recent developments and prospects in bile acid 7-dehydroxylation

An inverse relationship has been reported between *C. scindens* and DCA levels and *C. difficile* colonization (144). Several studies have incorporated *C. scindens* ATCC 35704 into small, defined consortia, providing insights into colonization, metabolism, and competition (145, 146, 147). In a murine model of *C. difficile* infection, addition of *C. scindens* ATCC 35704 was shown to have minor effects on the composition of the “Oligo-MM12” consortium but significantly decreased early colonization and pathogenesis of *C. difficile*, which was able to overcome colonization resistance by 72 h postinfection with concomitant decline in DCA levels (145). The inhibition of *C. difficile* strains by DCA-producing clostridia in vitro is reported to be strain-dependent, and *C. difficile* is ultimately able to outcompete these bacteria in defined communities (148). Secondary bile acids common in mice (eg, ω-MCA, HDCA, and MDCA) have also shown to inhibit *C. difficile* germination (149); however, human strains of *C. scindens* and other bile acid 7-dehydroxylating are not capable of biotransforming mouse bile acids (150). Eyssen reported isolation of a strain from rat feces that could 7-dehydroxylate murine bile acids; however, the phylogenetic position of this strain was not determined, and, to our knowledge, this strain was not preserved (150).

Recently, *C. scindens* G10 was reported to have been isolated from rat feces; however, only conversion of CA to DCA was tested (151). Future studies should determine whether strain G10, if indigenous to rats and mice, is capable of bile acid 7-dehydroxylation of muricholic acids. To further complicate this relationship, *C. scindens* ATCC 35704 was shown to generate tryptophan-based antibiotics that target *C. difficile* vegetative growth in synergy with the inhibitory effects of secondary bile acids (152). So far genetic manipulation of *C. scindens* ATCC 35704 has remained elusive, and determining the causal relationship between *bai* genes, antimicrobial peptide formation, and *C. difficile* colonization and pathogenesis remains to be determined (145, 153).

*C. scindens* DSM 100975 was recently isolated from pig feces and the complete genome sequenced (89). Comparative genomics between the human isolate ATCC 35704 and the pig isolate DSM 100975 share 2966 of 3655 genes, with the ∼675 genes unique to both strains composed largely of mobile genetic elements and phage genes. There is strict conservation of the *bai* gene cluster between the two strains, and in both cases, global transcriptomics revealed that the *bai* genes are significantly induced by CA, but not DCA (89, 94). It is not currently known whether this strain is able to convert hyocholic acid (3α,6α,7α-trihydroxy-5β-cholan-24-oic acid) to hyodeoxycholic acid (3α,6α-dihydroxy-5β-cholan-24-oic acid). The gnotobiotic piglet is a useful translational model, and the acquisition of *C. scindens* strains from different vertebrate species will allow future exploration into strain-specific adaptations to the gut environment.

Hayakawa and Hattori (1970) showed that *P. sordellii* NCIB 6929 produce DCA and 7-oxo-DCA from CA in vitro (154). Indeed, the first strains that were preserved in culture collections were *Paraclostridium bifermentans* ATCC 9714 and *P. sordellii* NCIB 6929 (154). A study in 1997 by Doerner *et al.* (86) compared the rate of bile acid 7-dehydroxylation activity from available isolates and separated the strains into two groups differing >100 fold in conversion rate of CA to DCA (86). *P. sordellii* is a “low” activity strain; however, it is unclear how this strain differs from “high” activity strains such as *C. scindens* or *P. hiranonis*. Possibilities include lack of a complete *bai* operon, changes in enzymatic or transporter activity, differences in promoter sequence or lack of inducibility, or perhaps other mechanisms exist beyond known *bai* genes. Indeed, we previously located two putative *bai* gene clusters in the *P. sordellii* ATCC 9714 genome consisting of *baiA_baiCD* and *baiH_baiE*, as well as a putative NADP-dependent bile acid 7α-HSDH (111). Predicted upstream regulatory proteins differ between *P. sordellii* (IclR) and canonical *bai* operons such as that found in *C. scindens* VPI 12708 (AraC). Could the *baiACDHE* genes (HSDH, 3-oxo- **Δ**^4^-reductase(s), and dehydratase) constitute an absolute minimal set of structural genes necessary for the conversion of CA to DCA?

Only a single study has reported the lack of 7-dehydroxylation in gnotobiotic gerbils colonized with defined consortium with or without *P. sordellii* ATCC 9714 (155). Another study noted a lack of DCA formation when bi-associating gnotobiotic mice with BSH-expressing *Bacteroides distasonis* and the 7-dehydroyxlating strains *P. hiranonis* TO931 or *Eubacterium* sp. strain 36S; the absence of DCA formation was explained by a lack of TCA conversion to CA (156). Successful deconjugation and 7-dehydroxylation of CA in this mouse model was reported after additional *Bacteroides* strains and *Bilophila wadsworthia* were added (157). In the *P. sordelli* colonization study, it was reported that 90% of conjugated CA and CDCA were deconjugated to free bile acids suggesting substrate limitation was not the issue (155). Further studies should examine the in vivo role of “low activity” 7-dehydroxylating bacteria to secondary bile acid formation.

A more perplexing observation from our genomic queries is the case of strains of *C. leptum,* which has been shown to convert CA and CDCA, but not UDCA, to secondary bile acids (79, 158). Conversion by this strain is known to be slow and inefficient (86). The rate-limiting bile acid 7α-dehydratase (BaiE) is in the SnoaL_4 family, and a search of BaiE from *C. scindens* VPI 12708 against the genome of *C. leptum* VPI 10900 revealed a single candidate with low sequence identity genome sequence of *P. sordellii* VPI 9048 genome that shares 48% AA sequence identity with the *baiE* gene product of *C. scindens*. Upstream of the putative *baiE* gene, we located an NADH-dependent flavin oxidoreductase with 47% AA sequence identity with the *baiCD* gene product and 64% AA sequence similarity with the *baiH* gene product from *C. scindens*. However, other *bai* gene candidates were not clearly observed. Perhaps this suggests a distinct mechanism by which this species converts CA to DCA. *Could this be the 7α-dehydratase and* ***Δ***^*6*^*-reductase of Bergström and Samuelsson*?

We think more likely that additional bile acid enzymes developed through convergent evolution from genes encoding members of the same or distinct protein families that serve analogous functions to those of canonical Bai enzymes. Indeed, Fe-S flavoproteins similar to BaiH and BaiCD are quite common, although their functions remain largely uncharacterized (159). Proteins in the short chain dehydrogenase/reductase, such as BaiA, are also widely distributed, and there are characterized 3ɑ-HSDHs (160, 161) distantly related to BaiA, indicating that additional examples will be found, perhaps in the genome of *C. leptum*. Further experiments will be required to resolve this issue, and *C. leptum* represents an interesting organism for future study in bile acid 7α-dehydroxylation.

### Overcoming barriers

A significant limitation in mechanistic gut microbiome research at present is the lack of genetically tractable model organisms, particularly in the *Firmicutes* (*Bacillota*). However, recent breakthroughs in *Firmicutes* (*Bacillota*) genetic tools are starting to make possible determination of the causal role of microbial genes in biochemical pathways, including bile acid pathways (128, 162) and the role genes/pathways in complex host phenotypes (163). *Faecalicatena contorta* S122 was recently identified as a low abundance (0.016% relative abundance) organism widely distributed in human metagenomes which harbors *bai* genes and actively converts CA to DCA (163). A genetic manipulation pipeline for nonmodel clostridia was developed in this study and knockout of the *baiH* gene in *F. contorta* S122 was achieved rendering the mutant incapable of in vitro conversion of CA to DCA. Stable colonization in both complex and defined communities in gnotobiotic mice treated with dextran sodium sulfate resulted in significant mucosal inflammation associated with *baiH*-dependent expansion of *Erysipelotrichaceae* in the gut (163).

## Conclusions

We hope that our decidedly and necessarily myopic historical focus will inspire others to obsess over the historical developments and *natural history* of similar biomolecules and most importantly, their relations to human health and disease. And we trust that such efforts will unearth as dedicated a cast of characters as we have attempted to outline in this story. Science is, after all, a human activity. With that being said, it is with great respect and admiration that we recognize Professors Phillip B. Hylemon and Ingemar Björkhem, with what, in our opinion, rightfully should be named after their dedicated career focus. This conclusion appeared to us obvious from our examination of the historical literature in bile acid 7-dehydroxylation and the foundation that preceded it.

There is a long precedence of naming metabolic schemes as such in the history of anaerobic bacteriology. Examples that are most famous include the Wood–Ljungdahl pathway of reductive acetogenesis attributed to the advances made by Harland G. Wood and Lars G. Ljungdahl (164) and the related Wolfe Cycle after the methanogen expert, Ralph S. Wolfe (165). In similar fashion, such scholars have had organisms named after them, such as *Acetobacterium woodii* (166), *Clostridium ljungdahlii* (167), *Methanothermobacter wolfeii* (168, 169), and in the present case, *Clostridium hylemonae* (81) and *Hylemonella* (170). The (unfathomably) ancient acetogenic and methanogenic pathways that utilize early Earth gases long predate vertebrates, and both pathways adapted to utilize the copious release of CO_2_ and H_2_ produced principally from carbohydrate fermentation in the gut of newcomers to this planet. But they have also adapted and evolved in the elbow-room-only environment of the GI tract in which, relatively speaking, methanogens, acetogens, and bile acid 7-dehydroxylating bacteria are low abundance bricks in the wall, albeit, in many cases, keystone species in the arch. Indeed, Phil Hylemon and one of the authors (JMR) have proposed a potential link between these pathways (120, 171). In light of the perspective of the past and the present, we look forward to the bright future of bile acid-microbiome-gastrointestinal-hepatobiliary discoveries.

## Conflict of interest

J. M. R. trained in the laboratory of Phillip B. Hylemon. All other authors declare that they have no conflicts of interest with the contents of this article.
